# Supplementary material for: Captivated by thought: “Sticky” thinking leaves traces of perceptual decoupling in task-evoked pupil size
Source: PLoS One. 2020 Dec 9;15(12):e0243532. doi: 10.1371/journal.pone.0243532 (PMC7725397; doi:10.1371/journal.pone.0243532)
Supplement: Attachment — Submitted filename: Review_ Captivated by thought.pdf [file pone.0243532.s001.pdf]

Review

Captivated by thought: “sticky” thinking leaves traces of perceptual decoupling in task-evoked pupil size

The manuscript describes a study investigating the pupillometric and behavioral performance correlates of a category of thought dubbed "sticky thinking", a form of perseverative thought that it is hard to disengage from. I think that the study is interesting and there is value to study this particular type of thinking; I also liked the transparency with which the methods and analyses are described, and I think that GAMMs are very promising to describe this type of data. I also have some major and minor comments that, from my point of view, could help improve the paper.

My main comments are about the two central topics in the manuscript: sticky thought, and perceptual decoupling. Regarding sticky thought, I see the authors introducing this concept as a (somehow) new category of thought. However it is not very clear (especially in the Introduction) how it differs from other types of mind-wandering or self-generated thought, and how it relates to them. The whole mind-wandering literature is plagued with a problem of semantics: researchers in the field often use different words to refer to the same thing, or the same words for different things. I think that in this context, conceptual clarity is even more important. For how it is described in the Introduction, sticky thought seems greatly conflated with general mind-wandering: e.g., the descriptions in line 39 to 43 could easily be used for mind-wandering. Sticky thought also seems conflated with rumination, a negative form of perseverative thought common in psychopathologies like major depressive disorder or anxiety disorders. I think that, if such a new concept is to be introduced in the literature, it should be clearly defined relative to existing concepts: for example, the authors say that it is a form of rigid, and narrow-focused thought process that it is hard to disengage from, but how is it different from rumination? I imagine that the authors implicitly assume that the stickiness of thought is a category of thought that is orthogonal to the valence of the thought (so that it can also be not negative), but in this case "neutral" and "positive" sticky thought should exist. Is this the case? Is there research on this, or could the authors provide examples? As the debate on the categories of thought is an ongoing one (see Seli et al., 2018, TiCS; Christoff et al., 2018, TiCS), I think it would be helpful to better define the concept of sticky thought relative to other types of thought. Moreover, for example the evidence provided in lines 46-54, or lines 65-67, is all related to rumination/worry, but the authors’ conclusions generalise to sticky thoughts: this does not seem warranted. While the idea of a “sticky” factor of thought is interesting, as it is now, I was not 100% convinced that it exists outside of rumination-like thoughts.

My other main comment is on the concept of perceptual decoupling. This refers to a hypothesized process by the brain, aimed at insulating the mental stream of thought from external (perceptual) distractions. It is an interesting hypothesis, but it is very much open to debate if such a process exists in the brain, and evidence is still limited. One previous study (Smallwood et al., 2011, Plos One) somewhat linked this hypothesized process to a reduction in pupil sizes during on-task and off-task periods of a sustained attention task; however, this is far from proving that small pupils are an index of perceptual decoupling.

While reading the manuscript, I feel that the authors imply that, finding smaller task-evoked pupil responses, automatically signals a perceptual decoupling process in action (e.g. lines 145,146, or 627-632). This seems like a form of reverse inference to me. The fact that perceptual decoupling (if it exists) might be linked to smaller pupils, does not necessarily mean that finding small pupils means that the brain is decoupling from the external environment. If this hypothesized process is really in act, we should observe other concurrent measures of reduced processing of the external environment: I don't think that the current paradigm in the study allows to do that, and additionally, there appears to be evidence for no differences in behavioral indexes of RT and RT variability between sticky and non-sticky thinking (e.g., line 623). While the stickiness factor did discriminate in no-go accuracy, this is a finding also common for mind-wandering thoughts in general, and is not intrinsically linked to perceptual decoupling. It is also not clear what the mechanism that would link smaller pupil task-evoked responses to a perceptual decoupling process would be. All in all, I do not think that the current study provides enough evidence to warrant strong affirmations such as those in lines 627-632, or lines 686-688. I don't know if the authors agree, but my suggestion would be to use a much more cautious language throughout the manuscript.

Some other comments that I have that I hope the authors will find useful:

- Lines 58-84: This is true but obviously not limited to sticky thought, but is one of the main obstacles in the field of mind-wandering and of consciousness research in general. I feel like the whole paragraph could be shortened if the authors have an interest in doing so, given that thought probes are pretty much the standard way in the field to study these types of ongoing thoughts.
- Lines 93-95: I have read the cited study and I couldn’t find some of the results here described (e.g. off-task thought, but not stickiness specifically, seemed to be related to task accuracy). Could the authors double-check? My apologies in advance if I missed or misread something.
- Line 97: One key reference that could be added here is Seli, Cheyne & Smilek (2013), JEP:HPP.
- Lines 141-142: This is slightly misleading as sticky thought is a novel concept. I do believe that there is some pupillometric research on rumination (e.g. Siegle, Steinhauser, Carter, Ramel & Thase, 2003), which could be added here, if the authors think it would be interesting for their argument.
- Lines 147-149: I was confused by this sentence, as it is possible to dissociate, and measure separately, baseline to task-evoked pupil responses. Could the authors clarify?
- Lines 149-151: Another study that could be discussed here and potentially in other parts of the manuscript is Konishi, Brown, Battaglini & Smallwood (2017), in which the authors find smaller baseline pupils for off-task thought, and specifically for negatively valenced and intrusive thoughts. As an author of that study I have a conflict of interest in pointing to it, but it seems to have some obvious links to the present manuscript (e.g. the concepts of intrusive and negative thoughts seems very close to those of rumination/worry).
- Lines 154-158: Indeed both smaller and larger baseline pupil sizes have been found in the literature (e.g. Gilzenrat, Niewenhuis, Jepma, Cohen, 2010; Smallwood et al., 2011, 2012, Van den Brink, Murphy & Niewenhuis, 2016; Van Orden, Jung & Makeig, 2000; Konishi et al., 2017; Unsworth & Robison, 2016), and it is still not 100% clear what factors account for these differences.

- Lines 163-165: This seems a little bit like a filler sentence. Maybe some concrete examples could be provided.
- Line 166: a “which” seems to be missing in the phrase “in we embedded”.
- Lines 170-174: To me it appears that these sentences construct a false dichotomy between sticky thoughts and general off-task thought/mind-wandering. For example, all the predictions described here can relate to off-task thought too.
- Lines 182-183: How was the number of participants decided?
- Lines 231-232: I think I missed how the experiment included 16 personal concern triplets, if the authors selected the 2 main concerns for each participant, and then translated each into a triplet of words. Sorry in advance if I misunderstood.
- Lines 249-251: Was this second question always presented, even if for example, participants reported to be on-task or externally distracted in the first question? Were the authors still analysing such cases?
- Figure 2: There’s a small typo in the third question (maters instead of matters).
- Line 263: A reference could be added for PsychoPy (the most recent study is Peirce, Gray, Simpson, et al., 2019, Behavior Research Methods).
- Line 304-305: It seems like the fixed order would cue participants to know in advance when a thought-probe would be presented. This could be seen as an issue, do the authors have any opinions on this?
- Lines 322-324: How were these cut-offs decided?
- Lines 364-365: This decision seems a bit strange in the context of pupil size and arousal, as external distraction is likely arousing, the opposite of inalertness.
- Lines 367-368: What was the reason? What was the point of including a 6 point scale if it’s not used in the analyses?
- Lines 481-483: Apologies in advance as I’m not sure if I’ve missed it, but was this result also taking into account the overall proportion of mind-wandering reports? If not, this result would be confounded by that, as they also increase over time.
- Figures 5 and 6: The legend for the plot on the right could be a bit clearer (or is missing).
- Lines 511-512: There is a serious and common problem in these designs, in which a thought-probe is presented always after a target stimulus. Following a mistake, participants might confabulate and report they were not on-task. This is a hard problem to overcome, but I think that this possibility should be discussed, given that the whole field relies on self-reports.
- Lines 516-519: Again, I am not 100% sure if I missed this, but was this analysis also taking into account the underlying proportion of mind-wandering thoughts?
- Lines 534-535: Did the authors analyse baseline pupil sizes across attentional states with simpler models such as LMMs? Given the amount of previous research on this, I’m just wondering if the null result depends somewhat on the GAMMs.
- Lines 565-567: It seems a bit strange that the difference is smaller between sticky and non-sticky than between sticky and neutral. If these are opposite of a continuous state, one would expect the difference to be bigger between sticky and non-sticky. Do the authors have any comments on this?

- Figure 10: The legend here could be clearer. Maybe it’s better to use colored lines instead of different patterns. It could also be that it wasn’t very clear because the image included in the review was lo-res.
- Line 654: Minor typo, missing a “to” in “compared neutral/non-sticky”.
- General: Would it be possible to have a table or a description of the best fitting models for each analysis conducted?
- General: Did the authors measure and check the response times to the thought-probes? It sometimes happens that some participants will start to respond very quickly to those probes (because they want to finish the experiment faster). Such fast responses should be discarded, as it is debatable if the participants can introspect and report on their previous mental states so quickly.
